# Standard employment and segmentation practices within the automotive industry in South Africa

**DOI:** 10.3389/fsoc.2025.1621307

**Published:** 2026-01-16

**Authors:** Irene Dingeldey, Andrea Schäfer

**Affiliations:** Collaborative Research Centre 1342 “Global Dynamics of Social Policy”, University of Bremen, Bremen, Germany

**Keywords:** automotive industry, collective bargaining, global production networks, labor law, labor market segmentation, non-standard employment, power resources, South Africa

## Abstract

Focusing on segmentation practices in an automotive manufacturing cluster in South Africa allows us to present a detailed picture of a specific industrial labor market in the Global South. Segmentation practices are outlined in terms of the variation in employment security and quality, encompassing the use of different types of employment, wage levels, and working hours. To explain these practices in collective bargaining and at the firm level, we draw on the insights of the global production network approach, as well as the labor market segmentation approach, which emphasizes national institutional settings such as general labor law, collective bargaining systems, but also power resources of different actors. Using a deductive qualitative design, we subjected 17 semi-structured interviews conducted in 2023 to qualitative content analysis and thematic analysis. This qualitative inquiry was further supplemented with quantitative data for the period 2022 to 2025 on labor norms and collective agreements, integrated within a theory-driven analytical framework. Although we find that South Africa formally adheres to a universalist labor law regime, opportunities for flexible forms of employment persist and are actively utilized within the sector. In addition, the bifurcated bargaining structure constitutes a key mechanism for segmentation. The structural power asymmetry between lead firms and suppliers is effectively transmitted to the workforce and its representation structures. This is reflected in substantially lower wages, longer working hours, and reduced employment and income security for workers at suppliers compared to those employed by lead firms. These findings demonstrate that, despite the presence of a universalist labor law regime, the power asymmetries between employers within the production network and along the supply chain as well as the bifurcated bargaining structure influence power relations between unions and employers and reproduce the distinctly segmented labor market practices in the South African automotive manufacturing network in Gauteng.

## 1 Introduction

The automotive industry is known to create high quality jobs around the world. It therefore has been regarded as a kind of ‘enclave’ (see Sturgeon and Florida, 2000, p. 86) in the Global South where national labor markets are usually characterized by high underemployment and considerable shares of informal work. Although we know that the fragmentation of production processes is associated with various elements of segmentation and stratification of the workforce, from a perspective of labor process theory Hammer and Riisgaard (2017) criticized the fact that this is not sufficiently reflected in the discourse and empirical research on global value chains (GVC) or global production networks (GPN). They highlight that only Lakhani et al. (2013) have addressed labor segmentation practices and how they are influenced by inter-firm relations and employment relations or that only Rainnie et al. (2011, p. 161) have explicitly addressed how actors’ agency and power resources can influence labor market segmentation within production networks. More recently, however, Flecker (2024) as well as Roberts and Tran (2023) have presented empirical studies on these topics. Moreover, the GVC and GPN discourse has not sufficiently integrated institutional settings as explanations for labor segmenting practices, although the institutional segmentation approach offers a rich literature (Deakin, 2013; Mückenberger and Dingeldey, 2022). However, we build on the existing work and advance it by integrating institutional settings as explanations to labor segmenting practices.

The focus of this paper is to analyze segmentation practices^1^ in a regional production cluster of the automotive industry in South Africa as part of a Global Production Network (GPN). Our research is guided by the following questions: What are the segmentation practices in terms of employment conditions in the South African automotive manufacturing industry? and: How do the national institutional settings and actors’ power resources influence segmentation practices at different levels of interaction?

Segmentation practices are investigated in terms of the variation in employment security and quality, encompassing the use of different types of employment contracts and their duration, seniority rules, wage levels, and working hours (López-Roldán and Fachelli, 2021). Since we are looking for segmentation practices within a particular labor market that is shaped by the fragmentation of production governed by particular (dependency) relationships between the ‘lead firms’ and suppliers (Sturgeon et al., 2009), our focus goes beyond the typical insider-outsider divide where employers create a protected core and a flexible, disposable peripheral workforce to adapt to market flexibility, which was highlighted by early segmentation theory (Doeringer and Piore, 1971; Reich et al., 1973). In line with more recently developed institutionalist segmentation theory (Grimshaw et al., 2017; Deakin, 2013), we also consider the national institutional settings, such as labor law and the collective bargaining system as well as the power resources of key actors, namely employers and trade unions (Keune and Pedaci, 2020). According to this approach, inclusive bargaining structures and powerful trade unions limit employers’ freedom and thus also limit dualization by enforcing equal treatment and more security in all parts of the labor market (Meardi et al., 2021, p. 43). This argument, however, underestimates the differences in firms’ power resources within production networks, as laid out in the global value chain literature (Gereffi and Guler, 2010, p. 118), as well as the strong alignment between a firm’s position in the production network and how it manages its workforce (Lakhani et al., 2013). To highlight and integrate these different aspects, we use power resource theory (Korpi, 1983) as a connecting element for our explanatory framework, distinguishing the actors’ different power resources. For employers, these power resources are considered in relation to their position in the production network, and for workers, more precisely trade unions and shop stewards, the focus is on different levels of interaction — as outlined by Arnholtz and Refslund (2024a) and Schmalz et al. (2018). As suggested by Flecker (2024, p. 171), looking at the opportunities and constraints of the vertical relations along the value chain and considering the position of the workplace and the workers within a network provides new insights into workers’ power resources in relation to their employer’s position within the production network. Thus, combining insights from the different theoretical approaches, we expect that employers’ varying structural power resources within the production network also influence trade unions’ and workers’ power positions. We assume that these power differences — especially between lead firms and suppliers — support segmentation practices within the production network, but also within the firm, leading to a deterioration in employment security and quality between lead firms and lower tier suppliers — albeit framed by the national institutional setting of labor law.

To develop our argument, we begin with a more detailed discussion of the different literature strands (2). We develop our research approach (3) and outline the case selection, data, and methods (4). We present an empirical analysis of norms of labor law as a regulatory framework and discuss their possible impact on the power resources of actors in South Africa, as well as their segmenting function (5). Describing the particular structure of the automotive production cluster we highlight the interrelationships between Original Equipment Manufacturers (OEMs) and suppliers according to their power positions in the production network (6). Finally, we present collective bargaining structures as a segmenting practice (7) and analyze how different employers deal with demand fluctuations at the firm level (8). In the conclusion we summarize and discuss our findings on the South African automotive production cluster and the added value of combining the segmentation approach, the global production network approach, and power resource theory (9).

## 2 Literature review

The *labor market segmentation approach* (see Grimshaw et al., 2017; Peck, 1989) provides deep insights into both practices and patterns of segmentation and offers a variety of explanations. The original construct was employer-centered, highlighting employers’ overall decisions and their ‘need’ to respond flexibly to market fluctuations by creating different employment segments and providing different opportunities for employment security, pay, and access to career opportunities (Doeringer and Piore, 1971; Reich et al., 1973). The *dualization debate* (Emmenegger et al., 2012; Palier and Thelen, 2012) brought trade unions’ strategies into focus to explain the divide between standard and precarious employment, although without unanimous results (Meardi et al., 2021). More recently, empirical research has clearly shown that trade union strategies to challenge precarious working conditions are strongly linked to their power resources (Keune and Pedaci, 2020).

The *institutionalist segmentation approach* highlights the influence of national institutional settings: industrial relations systems and bargaining structures are seen as influencing wage inequalities (Salverda and Mayhew, 2009; for South Africa see Hayter and Pons-Vignon, 2018). More recently, the segmenting function of legal regulation (Deakin, 2013), namely legal exclusion from and gradations of employment protection — mostly exercised by regulating the Standard Employment Relationship (SER) and flexible forms of employment — has been assumed to be a crucial influence on segmenting practices (Mückenberger and Dingeldey, 2022; Mückenberger et al., 2025; Mückenberger and Schäfer, 2025). A broad variety of segmenting patterns emerges as a result of segmentation practice within a particular legal framework. These patterns vary according to the share of the SER as opposed to different forms of flexible or precarious, but also of informal work (Vosko et al., 2003; Barbieri and Cutuli, 2016) and have been outlined by country (for a recent summary see ILO (2016, 2018) and by sector according to capital intensity and industry scale (Peck, 1989). Finally, discrimination based on personal attributes such as race, gender, migrant status, age, and disability is indicated by the fact that these groups are more likely to be affected by precarious work and pay gaps.^2^ However, this approach has hardly addressed the influence of particular inter-firm dependencies within value chains or networks and their impact on segmentation practices. Here the GVC and GPN literature can provide deeper insights.

The theory of *Global Value Chains* emphasizes inter-firm relations, specifically the global connectedness of production (Noronha and D’Cruz, 2020). The GVC in automotive production is considered to be an extremely concentrated structure, with just a few giant firms dominating production globally — and exercising an extraordinary amount of power over smaller firms. Assembly often takes place close to end-markets. As a result, the automotive industry has developed strong regional patterns of integration and is typically clustered in one or a few regions within a country (Sturgeon et al., 2009). However, the specific governance relationships between lead firms or OEM and suppliers can vary, depending, for example, on whether there is close cooperation on the design of parts (relational governance), or whether parts are developed in-house and then put out to tender (market governance) (Sturgeon et al., 2009). According to Fernandez-Stark and Gereffi (2019, 58), governance structures involve authority and power relationships which dictate how financial, material, and human resources are allocated within the chain and flow through it. In line with this concept, the power asymmetries between buyers and suppliers within the value chains are discussed in more detail by Bair and Mahutga (2023). The assumed vertical ordering has implications for employment conditions,^3^ suggesting that the major automotive companies and first-tier suppliers score well on ‘decent work’ dimensions, while lower tier suppliers do not (Gereffi and Guler, 2010, p. 118).^4^ This finding has been confirmed by South African case studies (Monaco, 2019; Mashilo, 2019).

However, the conceptualization of production and distribution processes as being essentially vertical and linear has also been criticized by the *Global Production Network (GPN)* approach, which emphasizes the complexity of inter-organizational arrangements that more often resemble a web or network (Wright and Kaine, 2015, p. 486). Distinguishing between corporate, institutional, and collective power it is acknowledged that firms at lower tiers might also gain some autonomy “to give rise to employment systems that are jointly influenced by lead firms and suppliers” (Lakhani et al., 2013, p. 454). In particular, Flecker (2024, p. 161) states that “power relations between firms strongly influence labour’s power in different nodes in production networks.” This implies that the fragmentation of production is linked to different segmentation and tiering practices (performing the same tasks under different contracts) that affect the workforce: including a range of precarious employment contracts based on different production relations, as well as through the supply and management of labor through third-party labor contractors — not only in outsourced production units, but also in OEM production sites (Hammer and Riisgaard, 2017). Moreover, the GPN approach assumes that ‘strong’ states and labor (Lakhani et al., 2013, p. 465), i.e., legal regulation and workers’ agency (Rainnie et al., 2011, p. 161; Riisgaard and Hammer, 2011), but also international agencies may attempt to exercise countervailing power either directly on firms or indirectly on national governments (Henderson et al., 2002, p. 450).

Overall, the discussion on social upgrading, namely the improvement of workers’ rights and entitlements to improve the quality of their employment, highlights the relevance of the particular national context (Barrientos et al., 2011, p. 324; Teipen and Mehl, 2022, p. 114). The idea is that the industry-specific modes of value chain governance and national labor regimes mutually influence each other. Hence, the country-specific type of industrial relations system and sectoral conditions such as collective bargaining structures and trade union power, but also state influence and public enforcement are important to explain employment structures and therefore opportunities for social upgrading (Teipen and Mehl, 2022, p. 114). Although these studies neither address legal segmentation nor do they explain segmenting practices in detail, we may follow a similar analytical approach. As both segmentation theory and the GVC/GPN approach highlight the relevance of power relations, we use power resource theory as a connecting theoretical framework. Classical power resource theory has a class-based foundation (Korpi, 1983) and generally emphasizes the difference in structural power between employers and workers due to the ownership of the means of production and the commodification of labor (Esping-Andersen, 1990). Different types of power resources are distinguished, such as structural, associational, institutional, ideational, and coalitional (Arnholtz and Refslund, 2024a) or societal power resources (Schmalz et al., 2018), with the latter two being more or less combined. Arnholtz and Refslund (2024a) simultaneously address the power to do something (power to) and power in relation to others (power over). Thus they emphasize that it is important to “not only focus on the power resources available to one actor, but also on the resources available to other actors” (Arnholtz and Refslund, 2024a:11). Findings from existing studies on the South African automotive production network dealing primarily with workers’ power resources (Mashilo and Webster, 2021) may thus be enriched by studying their systematic interrelation with employers’ power positions and the effect on employment segmentation practice.

## 3 Research approach

We understand segmentation practices — especially those affecting employment security and quality — as the practical implementation of legal and institutional norms. Rather than viewing legal segmentation as a static set of rules, we see it as a dynamic framework that takes shape through organizational practices. This approach highlights how firms interpret, adapt, or even challenge broader legal concepts in their everyday operations. Our framework offers a dual perspective. First, it enables an understanding of normative legal segmentation, such as the SER, which is based on stable, full-time, contract-based employment in combination with seniority rights, and additional forms of less protected employment, for example fixed-term contracts or labor brokers. Second, it allows us to analyze how different standards are applied in practice — through the use of different employment contract types, but also within wage setting, working time regulation, or internal job hierarchies. This perspective positions segmentation practices as a bridge between formal institutions and real labor market outcomes. It reveals how firms and workers’ representatives exercise different forms of agency and power in reinforcing or undermining labor norms and (re-)producing standards. The approach illustrates how employers and workers’ collective organizations in the GPN interpret, adapt, or even question overarching legal concepts in their day-to-day business, thereby (re-)producing labor market segmentation.

To identify particular segmentation practices in the South African automotive industry we lean on López-Roldán and Fachelli (2021). To sketch the dimension of security, we refer to the use of different forms of employment, namely the SER in contrast to more precarious forms of employment such as fixed-term (duration of contract), part-time, or contract work (also: temporary agency work or labor broker). Segmentation practices in terms of the quality of employment are reflected in different levels of pay for similar tasks at the level of the company or sector. This is accomplished by practices concerning working time (length of the regular working day, shift systems). The segmentation approach has generally addressed the impact of such segmentation practices on the division of the workforce within the firm or at the national level, highlighting the impact on different groups of workers according to gender, ethnicity, age. However, our focus is on how these practices affect the security and quality of employment — on how these segmenting practices vary within the production network and on how they are influenced by labor law and actors’ power resources at different levels.

Drawing on the literature, we consider the governance and fragmentation of the (global) production process and the complex relationship between lead firms and suppliers, or the position of the respective country and/or firm in the global production network, as a kind of sectoral specificity that is briefly sketched as ‘context’ since our analysis is directed at a regional production network of the automotive industry in South Africa. Insights from the GVC/GPN approach in combination with segmentation and power resource theory are used to analyze the interrelationships between different actors at different levels to explain the emergence of segmenting employment practices: first, between lead firms and suppliers; second, between employers or their associations and workers or trade unions within (sectoral) collective agreements, and third, between management and workers or their representatives at the firm level.

Following the literature (Arnholtz and Refslund, 2024b; Schmalz et al., 2018) we operationalize actor relations at different levels, drawing on different power resources with consequences for segmenting practices. Overall, structural power resources are assumed to differ not only between employer and employees, but also between firms according to their position in the production network. As we focus on actors who are more or less directly involved in determining segmentation practices, we do not analyze the ideational and coalitional or societal power resources used to influence political decision-making in detail. The role of the state is reflected only briefly in relation to certain elements of industrial policy with more or less direct consequences for the structure of the production network or OEM–supplier relations. In addition, we assess the type of national labor law as an institutional setting in terms of its dual function: the strength of direct legal segmentation with respect to employment relations, and the general framework for enabling or constraining actors’ options—thus defining institutional power resources (see section 5).

*The relation between lead firm and suppliers* can be described by differences in structural power resources that—according to the GVC approach—are ideal-typically derived from the ability to influence the activities of other firms, i.e., defining the products to be produced by suppliers and specifying processes and standards to be used. This form of structural power is exercised through the lead firms when subcontracting, meaning that entry to and exit from the production network is conditionalized and highly competitive (Gereffi et al., 2001, p. 4). Since for suppliers at lower tiers without any particular market power cutting labor costs is the cornerstone of competition, they probably would have to use practices such as flexible labor and lower pay compared to OEMs or higher level suppliers (Noronha and D’Cruz, 2020). Hence, a lack of structural power in inter-firm relations may encourage such employers to use structural power against their employees by implementing worse employment conditions. But awarding orders could also be related to monitoring suppliers with respect to quality standards. Some suppliers produce key components and can sell them to several OEMs, giving them a structural power position that allows them to better resist such demands—as suggested by the GPN approach (Henderson et al., 2002). Therefore, we examine whether and how employers in the first and second tiers justify segmenting practices to meet such demands.

As outlined in the literature review, the *employment relationship between the employers and the workers* is structurally imbalanced, with employers having greater power due to their control over production, while workers must sell their labor to secure their livelihood (Arnholtz and Refslund, 2024b). However, workers’ structural power—derived from their indispensability to the production process—can be leveraged to influence core aspects of employment security and quality, particularly through collective action such as strikes (Schmalz et al., 2018). This power is unevenly distributed among employees and shaped by labor market conditions, workers’ skill levels, and their functional role within production. Workers in permanent positions with high qualifications and central roles in firms with strong market positions are more likely to secure favorable employment conditions—such as open-ended contracts, wage progression, regulated working hours, and protection through seniority rules (Pulignano et al., 2016; Flecker, 2024).

The enactment of workers’ structural power is contingent upon organizational or associational power. Where union density is high and internal cohesion strong, workers are more capable of collectively negotiating employment standards (Schmalz et al., 2018). Trade unions and workplace representation bodies can influence contract standardization, limit the spread of precarious short-term or part-time contracts, and promote the extension of seniority-based protections (Flecker, 2024). At the same time, existing or increasing labor market segmentation weakens unions’ power and capability to do this, as precariously employed workers—often excluded from seniority schemes or standard contracts—are less likely to unionize (Keune and Pedaci, 2020). This necessitates inclusive bargaining frameworks beyond traditional union structures to counter employers’ use of differentiated contract forms as a means of workforce segmentation (Benassi and Dorigatti, 2015; Doellgast et al., 2018). Such frameworks can reveal whether power is used to entrench dualisms — preserving favorable conditions for core workers while neglecting others — or to promote uniform standards in employment security and quality across the workforce.

Employers, in turn, may strategically leverage their structural power to shape employment conditions. Larger firms with stable market positions may offer more secure contracts and better working conditions, as they require a stable, skilled workforce. In contrast, smaller firms or those facing competitive pressures more often rely on temporary or flexible contracts with limited employment protection. Diverging interests within employer associations — between firms favoring secure employment standards and those pushing for flexibilization — may lead to organizational fragmentation and/or loss of associational power on the employer side (Alsos and Evans, 2018). Additionally, the existence of non-unionized firms can serve to undercut negotiated standards by applying cost-cutting practices such as contract casualization, variable working hours, and wage suppression.

*Institutional power* plays a crucial role in regulating employment security and quality. Legal instruments such as limits on fixed-term contracts, seniority-based dismissal protection, statutory wage floors, and working time directives enhance employment security and reduce employers’ unilateral control (Deakin, 2010). Legal frameworks that guarantee freedom of association, regulate collective bargaining, and enforce co-determination rights at the workplace directly increase unions’ power resources and thus indirectly have an influence on the use of different employment contracts, their duration, and conditions. Furthermore, the extension of collective agreements and legally-backed union incentives can mitigate fragmentation and promote inclusive employment standards across sectors.

As we have argued so far, we understand segmenting practices in the context of employers’ power resources. These are determined by their position within the production network and the distribution of power resources within the employer-employee relationship at different levels of interaction. Additionally, we assume that legal regulation may itself have segmenting functions, yet still can restrict employers’ scope for direction and/or support trade unions’ power resources.

Since our approach aims to understand segmentation practices in relation to structural, institutional, and organizational positions of power held by employers and trade unions, it has also some limitations. It falls short in explaining segmenting practices related to social practices and discrimination. Hence, we only touch briefly on how different groups of workers, for example according to ethnicity or age, are affected by different forms and quality of employment and the explanation of these practices unfortunately lies beyond this approach.

## 4 Case selection, data and methods

OEMs in South Africa are located in several major industrial hubs, of which the Gauteng province is the largest. Gauteng is home to the Ford Motor Company of Southern Africa (in Silverton/Pretoria), the Bavarian Motor Works (in Rosslyn), Nissan South Africa (in Rosslyn), and has a high density of vehicle component manufacturers (Mashilo, 2019). As an expert from the National Association of Automotive Component and Allied Manufacturers (NAAMSA) noted “*Gauteng is typically the economic hub of the country. The three OEMs here, BMW, Ford, Nissan, and the majority of the biggest growth of the country’s component sector is in Gauteng.*” (Interview_NAAMSA). The strategic location of the OEMs in Gauteng ensures their close proximity to their suppliers and to Transnet Port Terminals also through the support and establishment of the Tshwane Automotive Special Economic Zone (SEZ), Industrial Development Zone (IDZ) and Automotive Supplier Park in Rosslyn Pretoria.^5^ An expert from NAACAM added: “*The Gauteng autos economy is really, I think, larger and diverse by virtue of having three of the OEMs assembling vehicles here, at least from a light motor vehicle perspective. Those three are Nissan, Ford and BMW. And they would have their own supplier networks and the supplier base set up.*” (Interview_NAACAM).

Between October 2022 and May 2023, we conducted 20 semi-structured interviews with key stakeholders in the Gauteng regional production network, including trade union officials (at both plant and regional level), respondents from the employer side (managers in OEMs and suppliers), and representatives from employers’ associations. The interviews were conducted either face-to-face at company or union offices (11 interviews) or via video call (9 interviews) and lasted approximately 55 to 85 min each. All interviews were audio-recorded and transcribed for analysis. Interviewees were guaranteed anonymity and are referred to in the findings using generic identifiers. We used a semi-structured interview format, which allowed interviewees to discuss their experiences freely while ensuring that certain core themes were covered. These were: (1) position in the production network —the role of the company or union in the cluster and relationships along the supply chain. (2) Employment relationships — the nature of jobs, employment contracts, and workforce composition in the firm or sector. (3) Use of non-standard employment — the types of atypical or flexible contracts used (e.g., temporary, part-time, subcontracting) and the practices for managing them in the firm/cluster. (4) Compliance with and enforcement of labor law and (5) ongoing transformations. The interviews were iterative; questions were adjusted as new insights emerged. We followed the principle that qualitative research should be “open to the new in the analyzed, the unknown in the seemingly known” (Flick, 2013, p. 17).

The final sample for this article comprised 17 interviews with 24 participants, including eight union representatives, 12 employer representatives from one OEM and multiple supplier firms (tier 1 and tier 2), and four employer association officials (Table 1).^6^ As shown in Table 1, we used purposive sampling to recruit participants from all the main stakeholder groups in South Africa’s automotive industry to ensure that their voices were represented in the data, providing a broad range of labor and management perspectives at different organizational levels — a crucial consideration when analyzing labor market segmentation in a context of strong labor regulations and corporatist industrial relations. Interviews were conducted until data saturation was reached and no new themes emerged with the addition of each subsequent interview. Reaching saturation after 17 interviews indicated that the sample size was sufficient for reliable qualitative analysis (Hennink and Kaiser, 2022, p. 9).

**Table 1:** Interview participants by organization and function, 2023, Gauteng region.

Organization	Number of interviews	Number of interviewees	Interviewee’s function within organization
Union	7	8	Shop stewards, national coordinator, legal adviser
Employer			
OEMs	1	1	CEO, HR officer/head, owner, regional leader
Tier 1	5	9	
Tier 2	2	2	
Employer associations	2	4	Project manager, executive trade rep., executive director, executive HR
Total	17	24	

Sources: Authors’ description.

Additionally, we draw on the CBR Leximetric Datasets, Version 2, from the Centre for Business Research at the University of Cambridge (Deakin et al., 2023), supplemented by data from the World of Labour project (see Carlino et al., 2024; Carlino et al., 2025), to compile a dataset comprising numerical indicators for individual and collective labor law norms for South Africa in 2022 (see Table 2). Trade union documents (e.g., internal reports) and collective bargaining agreements (provided by NUMSA) were used to map formal regulations and entitlements in order to assess segmentation practices.

**Table 2:** Indices and indicators of legal norms for employment in South Africa, 2022.

Indicator on national individual or collective labor law regulation	Value
Labor Rights Index (LRI)^1^	79
Index of protection function^2^	0.69
Index of privileging function^2^	0.23
Index of equalizing function^2^	0.85
Index of flexibility function^2^	0.31
Index of employee representation in labor laws^3^	1.00
Index of codetermination^4^	0.34

Sources: Authors’ own calculations and descriptions using CBR-LRI Version 2 (2023) at Doi: 10.17863/CAM.9130.2 and complementary data from WoL (see Carlino et al., 2024; Schäfer, 2024a,b,c; all indicators to be found in data repository: www.wesis.org), ILO at https://ilostat.ilo.org/data/data-catalogue/. ^1^Statutory labor rights for the workers in formal employment relationships from ILO data. ^2^WoL indicators are coded using statutory law only (Carlino et al., 2025), CBR-LRI data uses statutory law or collective agreement for coding data; Regarding WoL: Some indicators use binary coding but most use non-binary or graduated scores. ^3^Includes right to unionization, right to collective bargaining, and extension of collective agreements from CBR-LRI Version 2 (2023). ^4^Includes codetermination and information/consultation of workers and codetermination: board membership from CBR-LRI Version 2 (2023). LRI: 0 = “Total Lack of Decent Work” to 100 = “Decent Work”; all other indices: ‘0’ stands for no protection/ privileging/ equalizing/ flexibility or the lowest protection offered to workers, and ‘1’ stands for the maximum or highest protection/ privileging/ equalizing/ flexibility offered.

Adopting a deductive qualitative design (Fife and Gossner, 2024), we conducted a qualitative content analysis (Mayring, 2015) and thematic analysis (Braun and Clarke, 2006) of the semi-structured interviews. Mayring’s rule-guided procedure enabled transparent and reproducible coding according to a predefined, theory-driven codebook. Braun and Clarke’s approach synthesized patterns across cases, clarifying the thematic structure. We analyzed three *a priori* themes: (1) the firm’s economic situation and its consequences for non-standard work, (2) dependency relations (on OEMs and other firms) and their consequences for non-standard work, and (3) union influence and power, and related consequences. Segmentation practices were classified transversally: security via contract forms (SER vs. fixed-term, part-time) and quality via pay differentials shaped by working time regimes (hours and shifts). The analysis was iterative; when new cases no longer yielded new themes across OEM, tier-1 and tier-2 settings, and deviant cases fit as variants, theoretical saturation was indicated without expanding the codebook. Simple triangulation using legal/tariff documents and sectoral data supported the findings. This dual approach provides methodological rigor and cross-case interpretive depth, which is aligned with our theory-led research question on segmentation in a tightly regulated context.

## 5 Segmentation in labor law and power resources

As will be discussed, South Africa has a strong and inclusive labor law with strict regulations on non-standard employment (Table 2). In post-apartheid South Africa, the Labour Relations Act 66 of 1995 (LRA)^7^ provides workers with full collective rights: freedom of association and collective bargaining, allowing workers to organize and negotiate binding agreements with employers, but also full legal backing for employee representation in the workplace, which signifies high institutional power resources for workers. This is reflected in the highest index of workers’ representation in labor laws (1.00), although co-determination (0.34) shows limited employee participation in decision-making processes, such as board membership and consultation rights (Table 2). Additionally, legal extensions to collective agreements, such as the agreement with the *Motor Industry Bargaining Council* (see below), equalizes employment conditions across the industry. The law also allows unions to enter into an agreement with the employer to charge non-unionized employees a bargaining fee (see National Bargaining Forum agreement) that may not be higher than the union membership fee. This can be seen as an incentive to join a union that provides additional services to its members. Both features thus provide legal support for unions’ associational power resources.

Despite these general rights, there is legal segmentation within South African labor law. Coverage by agreements is generally limited to workers earning less than the National Wage Threshold, which is specified under the Basic Conditions of Employment Act by the Minister of Employment and Labour (P.211 Gazette; it was R 20,092.54 per month in 2024^8^). This limits the scope of the industry’s collective agreements to (mostly Black or Coloured) blue-collar workers. Conditions of employment and wage adjustments for white-collar workers are determined in company-specific agreements (Mashilo, 2022, p. 241). To establish workers’ representatives on the shop floor, a trade union must prove that it organizes the majority of workers in the workplace and then the representative has to be elected by at least ten trade union members, meaning that small companies are excluded from workplace representation. The labor law thus creates different levels of protection for workers depending on their income and occupational position and limits collective associations on the basis of firm size.

The Labour Relations Act 66 of 1995 (LRA) provides strong individual protections to enhance employment security. The LRA states that dismissals must be for a fair reason and in accordance with a fair procedure. These provisions protect against arbitrary dismissal and create more secure, longer-term employment. Seniority is recognized in law and practice as a factor in employment security —especially with respect to employees with SER contracts. In the event of redundancy, the law provides for increasing notice periods based on seniority, from one week for short service to four weeks for over a year’s service, and requires at least one week’s severance pay per year of continuous service. Moreover, employers must consult with unions or workers and try to avoid or minimize redundancies. If redundancies are unavoidable, the selection criteria must be fair and objective; the common ‘last in, first out’ (LIFO) system often protects longer-serving employees. While skills and performance can also be considered, the frequent use of LIFO structures dismissals in a way that values seniority, thereby strengthening job security for long-term employees — and enacting legal segmentation. In addition, the Basic Conditions of Employment Act 75 of 1997 (BCEA)^9^ regulates working hours and leave, ensuring predictable work schedules. The BCEA limits the normal working week to 45 h (with a maximum of nine hours per day in a five-day week) and requires premium pay for overtime and work on Sundays or public holidays. Legislative reforms have specifically targeted precarious employment contracts to improve employment security. Employees on fixed-term contracts are entitled to the same treatment as permanent employees. Moreover, the LRA (as amended in 2014, Section 198B) deems a fixed-term contract to be permanent if its duration exceeds three months without justification or when a succession of fixed-term contracts lasts more than 24 months. Similarly, after three months, Temporary Employment Service (TES) workers are considered employees of the client company with permanent status in order to prevent the circumvention of fair labor standards. Additionally, and as a result of union pressure, a national minimum wage was introduced in 2018 — to fight creeping job insecurity and support workers at the lower end of the pay scale (see Hayter and Pons-Vignon, 2018).

Although some forms of legal segmentation exist, for example to protect employment security according to seniority, and to regulate workplace representation, South African labor legislation strongly restricts employers’ scope to use flexible forms of labor. Additionally, it provides rather extensive institutional power resources to support union organization, workers’ representation at the shop floor, and it encompasses collective bargaining. Assessing South African labor law by quantitative indicators, rather high scores for the protection function (0.69) and low scores for the flexibility function (0.31)^10^ confirm that the South African legal framework tends towards stability and protection for workers. A low score for the privileging function (0.23) combined with a high score for the equality function (0.85) indicates the promotion of equal treatment not only according to gender, but also according to different forms of employment contracts (see Table 2). Thus, the different regulations of both collective and individual labor law add up to a moderately strong, inclusive, and protective type of labor law with respect to both collective (see also Hayter and Pons-Vignon, 2018) and individual rights (for 2013 see Mückenberger and Dingeldey, 2022). How this influences segmenting practices in the automotive sector is explored in the following sections.

## 6 Automotive production in South Africa—segmentation and structural power relations within the production network

The origins of vehicle manufacturing in South Africa can be traced back to 1924 (Mashilo, 2022, p. 229; Lamprecht, 2024). In 2022, a total of 309,423^11^ passenger cars were produced in South Africa, representing a 0.65 percent share of global vehicle production (Naamsa, 2025, p. 2). However, although the global impact of the South African automotive industry is small, it is one of the most advanced manufacturing hubs on the continent. Within the domestic economy, it accounts for 21.9 percent of manufacturing value added in 2023 and contributes 5.3 percent to GDP (see Table 3). Exports of vehicles and vehicle components to 148 destinations account for 14.7 percent of South Africa’s total exports—all of which are growing in importance.

**Table 3:** Main economic indicators on the automotive industry in South Africa, 2021–2023.

Indicator / Year	2021	2022	2023
Broader automotive industry contribution to GDP (in percent)	4.3	4.9	5.3
Vehicle and component production as share of South Africa’s manufacturing output (in percent)	17.3	21.7	21.9
Automotive export revenue as share of total South African export revenue (in percent)	12.5	12.4	14.7
Automotive component sector employment (total)	78,874	83,362	82,560
Average monthly employment by vehicle manufacturers (total)	30,697	33,321	33,509

Source: Author’s compilation using Lamprecht, 2023a, 2024, p. 5, 6.

The South African automotive industry is a compressed and underdeveloped three-tier production chain (Barnes et al., 2018, p. 6/7). In 2022/23, seven international OEMs were supplied by an estimated 198 tier-1 suppliers specialized in vehicle systems, modules, and subassemblies. The 200 tier-2 suppliers manufacture the components and subcomponents that are incorporated into complete vehicle systems, modules, and subassemblies. Tier-3 suppliers provide raw materials (Lamprecht, 2024; Mashilo, 2022, p. 231). While 75 percent of the top-tier suppliers are foreign multinational corporations, South African firms are found in the second and third tiers (Lamprecht, 2024, p. 79)—a structure underlined by a representative of the suppliers’ industry association NAACAM: “*We are very much tier one heavy, that is the predominant grouping of manufacturers. And then we have a much smaller tier two and tier three base.*” (Interview_NAACAM).

This structure emerged due to post-apartheid industrial policy when in 1995 the lifting of international sanctions was accompanied by a new automotive industrial policy that gradually reduced the industry’s protections to encourage increased foreign capital investment. Incentives to increase production volumes were provided, incentivizing exports with tax-based import rebate credits allowing vehicle exporters reduced-duty or duty-free imports of the components they did not source. Consecutive South African governments launched development programs aiming to improve local production within the automotive industry, of which the latest is the South African Automotive Masterplan (SAAM) of 2021. So far, South Africa’s post-apartheid industrial policy has been successful in terms of significant growth in the value of its vehicle assembly activities (from R 75 billion^12^ in manufacturing sales to R 137 billion between 2012 to 2015). But this was accompanied by a R 44 billion surge in automotive component imports over the same period, largely nullifying the large assembly gains made (Barnes et al., 2018). In 2012, value addition by OEMs located in the South African automotive industry was significantly higher (40 percent) compared to other countries globally (20 percent). For tier-1 suppliers, the relation was 40 to 30 percent. Accordingly, for second- and third-tier suppliers in South Africa, value addition was significantly lower than elsewhere (only 20 compared to 50 percent) (Monaco et al., 2018).

This distribution of value added can be seen as the result of substantial structural power differentials between international OEMs and tier-1 suppliers and (local) tier-2 suppliers in South Africa, which may influence the practice of employment segmentation in terms of wages. However, specific relationships need to be explored, for example, dependence on a single supplier may be mitigated by the diversity of OEM customers in the region: “*Often you find that there are supplier companies who supply more than one OEM and especially in a, let us call it a smaller tier economy like South Africa, you will find many of the component manufacturers have multiple OEM customers*” (Interview_NAACAM). This is not to say that the general structural power imbalance between OEMs and suppliers is nullified since OEMs are still able to shape the regional production network: “*We do prefer that if there’s any sort of new suppliers coming on board […] that they station themselves within the [special] economic zone, because it makes it easier from an access perspective*” (Interview_JM).

An example of how the structural power of OEMs and tier-1 suppliers is used more or less directly in the production network is the implementation of national political demands for Black empowerment. With the SAAM, the government aims to increase local content of vehicles assembled in South Africa by 20 percentage points to become 60 percent by 2035. To achieve this goal, supply chain improvement and transformation and Black economic empowerment have become increasingly linked. Launched in 2003, the latest iteration of Broad-Based Black Empowerment (B-BBEE; short: Triple B) policy aims to significantly increase the number of Black people who manage, own, and control the country’s economy. Progress on B-BBEE performance is monitored using scorecards that assign values to (Black) ownership, management control, skills development, socioeconomic development, and enterprise and supplier development. As the measurement categories became mandatory, accompanied by increased reporting requirements, this created some pressure on international firms to transform, as B-BBEE certificates are essential to securing certain incentives or contracts with the state or with other private entities (for more details see Barnes et al., 2021; Vilakazi and Bosiu, 2021; Mashilo and Moothilal, 2022).

Already during the preparation of the SAAM, however, representatives of German OEMs—even from the home county — made clear that they would no longer have any rationale to produce automotive vehicles in South Africa if automotive industrial policy were to change and B-BBEE ratings were to become extremely stringent (Mashilo and Moothilal, 2022). This was interpreted by experts as “*The multinational OEMs determine the terms of business and engagements and they guide their respective subsidiaries under the overall strategic direction that they set from their headquarters*” (Mashilo and Moothilal, 2022). A new iteration of B-BBEE codes then foresaw that ratings monitor not only progress made in the certified firm, but also by its suppliers. Taking advantage of this final regulation, OEMs or international tier-1 suppliers now use their structural power to ‘encourage’ their suppliers to improve their scores. An OEM executive noted that they “*will not do business with anyone who does not comply with human rights [and] the labour legislation of our country*” (Interview_JM) and that they prefer suppliers with a good Black Economic Empowerment score. These policies are implemented via audits and incentives such as multi-year contracts or more stable ordering, but also by making clear that failure to meet the required standards could jeopardize the contract. This was mentioned by a tier-2 supplier, who had to substantially improve its B-BBEE score to continue doing business with a major multinational tier-1 customer. “*We were then level 8 [B-BBEE], they said, the minimum is level 6 and then they said I think 2019 or 20 we have to be a level four. So it was a concerted effort from the company*” (Interview_CR). The mechanism of delegating state-set responsibilities involves juggling demands from above (OEM) and ensuring performance of suppliers below—as described by the tier-2 supplier in the middle of the chain: *“So it’s actually a trade-off process where everybody tries to manage their suppliers. Otherwise you get penalized too much [depending on what your mixed score card is]*” (Interview_CR). In general, this confirms the GVC and GPN literature as well as our assumptions that structural power differences extend both downward (placing requirements on sub-suppliers) and upward (meeting OEM and tier-1 supplier requirements), forming a network of compliance obligations that cascade through the tiers. In this particular case, the use of structural power by lead firms and tier-1 suppliers to comply with government requirements with respect to human rights or B-BBEE goals across the value chain may contribute to improving employment conditions and enhancing equity — and thus counteracting segmenting practices. More often, however, OEMs’ structural power dominance is used to produce opposite effects when the focus is on reducing production costs (see particularly section 7).

## 7 Power resources of unions and employers: collective bargaining divisions as segmenting practice

Since national labor law provides freedom of association and collective bargaining as institutional power resources for trade unions, they could develop organizational power. The rapid growth of industrial unions representing Black lower-skilled workers was, however, confronted with a rather fragmented landscape of different union federations at national level. Tensions between the Congress of South African Trade Unions (COSATU), the largest federation, and the National Union of Metalworkers of South Africa (NUMSA) resulted in a split in 2014. NUMSA then became a member of the now second-largest South African Federation of Trade Unions (SAFTU), launched in 2017 (Hayter and Pons-Vignon, 2018). NUMSA is the leading trade union in the automotive sector, organizing primarily blue-collar, hourly-waged Black production workers (Mashilo, 2022): “*Well, NUMSA is one of the biggest, you know, trade unions in sub-Sahara with a membership of close to 400,000. We organize across the different value chains and different sectors of our society*” (Interview_GQ). Trade unions’ organizational density in the sector is assessed at approximately 39 percent (Mashilo, 2019, p. 48), compared to 19.1 percent nationally (ILO, 2025a, 2025b), indicating that the automotive sector is a stronghold of unions’ organizational power. NUMSA is potentially able to counter employers’ structural power advantage.

But organizational power and representation at the workplace in the automotive sector tend to be stronger at OEMs than at suppliers: “*In our country, almost 100 percent of OEMs are highly unionized. Most of the shop stewards are full time shop stewards.*” (Interview_GQ). Within the tiers, however, we do not see a uniform picture, but — according to our experts—rather individually-governed employment relations. While a tier-1 company described a “*very open and tight relationship with our union*” (Interview_CK) and formal recognition agreements with NUMSA as the majority union, a union representative in another tier-1 seat manufacturer claims to have “*a difficult employer*” that “*does not want to engage with the union … [the manager] does not believe that the two parties can resolve and come up with a better way to improve their production*” (Interview_GQ). A third tier-1 supplier even refuses to engage with unions at all, following a paternalist management style: “*No, I do not. Okay. So that rolls back to my deal of 20 years ago with the 16 staff that we had. We said, okay, we’ll only employ your friends and family on condition: no trade union. We have a closed shop, touch wood. We’ve never had a stoppage. We’ve never had a strike.*” (Interview_ANT). Moreover, the link between employment conditions and union representation at firm level is not fully clear, since the paternalist management style, for example, results in job security and pay in accordance with collective agreements.

However, general differences between OEMs and suppliers are underlined by two distinct associational entities representing employers’ industrial and collective bargaining interests. This results in the use of collective bargaining divisions as segmenting practices. OEMs promote the interests of their industry within The National Association of Automobile Manufacturers of South Africa (NAAMSA), whereas the Automobile Manufacturers Employers’ Organisation (AMEO) represents their interests as employers. AMEO and the union NUMSA bargain within the *Automobile National Bargaining Forum* (NBF). In the NBF, wages and other substantive conditions of employment for all South African OEMs are negotiated, ensuring uniform conditions across the country. One OEM expert describes the advantages from an employer’s perspective: “*we have one common union… and we negotiate at a national level. If you do not do that… you are going to have one OEM doing this, the other one doing that… it’s just going to create disharmony. …Negotiating centrally and implementing at an organizational level… minimizes labor disruptions*” (Interview_JM). Equal working conditions across different OEMs is also appreciated by a trade union representative: “*what is paid in Ford is also paid in BMW… you can never have lesser conditions [at one OEM than another*]” (Interview_VM).

The National Association of Automotive Components and Allied Manufacturers (NAACAM) is the industry body of the component manufacturers. The *Motor Industry Bargaining Council* (MIBCO) covers component manufacturing, vehicle body building, and automotive engineering and reconditioning establishments, and other branches, for example sales and fuel retail, on the national level. Here NUMSA negotiates with the employers’ associations the Retail Motor Industry (RMI) and the Fuel Retailers Association (FRA), agreeing on wages for different sub-sectors. Although NUMSA is trying to advance its strategy of centralized sectoral bargaining by establishing a single forum for automobile manufacturing industry-wide centralized bargaining — thus uniting the industry’s existing bargaining councils (Mashilo, 2022, p. 243) — they have not yet been successful. Hence, their structural and organizational power resources are obviously still not strong enough to successfully pursue an inclusive union strategy to equalize employment conditions.

The NBF and MIBCO agreements implement a sharp divide in employment quality in the form of pay and regular working time. But also within the MIBCO agreement we see differences across the different branches of suppliers—albeit at a much lesser scope (Table 4). Although skill levels in the two agreements may not be defined exactly along the same criteria, a comparison indicates that in 2023 the negotiated wages in OEMs were more or less three times higher than in manufacturing and even up to four times higher than in vehicle body building establishments—where the collectively negotiated minimum wage for skill level 1 was only slightly above the legal minimum wage of R 25.42 per hour in March 2023 (Department Employment and Labour South Africa, 2024). Although the differences decline slightly for the higher grades, they are still enormous. Additionally, OEMs pay one flat-rate gratuity and an annual gratuity related to earnings. As the yearly wage increase negotiated with OEMs is at least one percentage point higher than for the suppliers, the existing wage gap will further increase (Table 4).

**Table 4:** Description of employment quality criteria in collective agreements in South Africa, 2022–2025.

Kind of agreement / employment quality criteria	NBF Agreement on wages and conditions of employment	MIBCO Main collective Agreement; Chapter III: Manufacturing establishments	MIBCO Main collective Agreement; Chapter II: Vehicle body building establishments
Negotiated wage increase by year (in percent)	2022: 8.5 2023: 7.0 2024: 7.0	2023: 7.5 2024: 6.0 2025: 6.0	
Plus	R 10,000.00 (one-off taxable gratuity amount) a year-end gratuity of 8.33 percent of basic pay	None	
Minimum wage per hour in skill level^1^ (in Rand)^2^	in 2022/23	since August 2023	
1^3^	109.10	28.71	25.88
5	163.08	49.82	45.81
Highest: NBF:7; MIBCO:8	232.19	85.19	78.36
Ordinary working hours	40 (per week)	45 (per week) or 9 (per day)	
Overtime bonus	One and a half times the ordinary rate for the first 6 h, then double	One and a half times the ordinary rate (6–23:00 h); double (23–6:00 h)	
Short-time work compensation	50 percent of basic daily wage, maximum 50 days/ year	No pay	

Sources: Author’s own compilation using Composite National Bargaining Forum (NBF) (2022). Agreement on wages and conditions of employment applicable to all hourly-paid employees in the automotive manufacturing industry (for the period July 1, 2022 to June 30, 2025) provided by NUMSA; Motor Industry Bargaining Council – (MIBCO) Main collective Agreement (August 2023–August 2025). ^1^Level 1 according to NBF: Certificate 1 = 20 percent artisan module; Level 5: Artisan trade certificate; Level 7: Multi-skilled artisans / technicians. ^2^Exchange rate 2023: 1 USD = R 18.5 on average, see: https://www.dollarfx.org/South-African-Rand/2023. ^3^As point of reference: The legal minimum wage in March 2023 was R 25.42 per hour.

Another substantial difference in the collective bargaining agreements is regular working time: 40 h a week at OEMs and 48 h at suppliers—with an impact on when overtime premia have to be paid. Although the longer working hours may partly compensate for the gradation differences in monthly earnings, they are a major indicator for deteriorating employment quality between OEMs and suppliers — as commented on by a trade union representative: “*It then deals with the terms wages and terms and conditions of employment, which are somewhat better than others, which are only value chains. Going down the other value chains, then the conditions become lesser and lesser and lesser and lesser. But in the OEMs, especially those who are working directly in manufacturing vehicles, I can safely say they are better paid than the value chain. Yes.*” (Interview_VM).

Beyond pay and working time we find other substantial differences affecting employment security and quality: While OEMs compensate short-time work with at least 50 percent of regular pay, non-pay during short-time work is part of the MIBCO agreement (Table 4). Furthermore, the treatment of flexible work is also different: While in the collective agreement with OEMs, NUMSA achieved a ban on the use of temporary employment services since 2011, in the agreement with suppliers, the share of temporary employment services was only limited to 35 percent of the core workforce since 2013 (Mashilo and Webster, 2021, p. 547; see also MIBCO Gazette, 31 March, 2023, p. 62). Additionally, training and qualification measures and support for shop stewards’ work are only addressed in the agreements with OEMs. Employment guarantees and (social) benefits such as transport subsidies, medical aid, and provident funds are generally not the subject of negotiations with suppliers, and if they are, agreed conditions are at much lower level than at OEMs.

In South Africa, collective bargaining structures thus function as a segmentation practice to divide job quality and pay between OEMs and suppliers. This is mirrored by the organizational division of employers, which reflects different structural power positions within the production network as most suppliers can only obtain subcontracts through lower production costs, which are achieved through lower labor costs. Workers’ organizational and structural power resources seem to reproduce the segmentation lines between OEMs and suppliers, albeit with a great deal of variation according to the situation in individual supplier firms. These segmenting practices also extend to employment security matters, as demonstrated by the use of flexible work or short-time working practices on the shop floor.

## 8 Employment patterns and segmenting practices at the firm level

South Africa’s national labor market is characterized by more or less high unemployment (nearly 32.1 percent on average in 2023) (ILO, 2025b), strongly diverging along ethnic lines (about 36 percent for Blacks, 23 percent for Coloureds and only 9 percent for Whites) (Statista, 2025). This is accompanied by a rather low rate of informal work in 2023 (34 percent on average) — at least compared to other African counties (estimated average of 83 percent) (ILO, 2025b). In the automotive sector, a total of 116,069 workers were employed in 2023, about two-thirds in component manufacturing and one-third in vehicle manufacturing (OEMs) (see Table 3). Black workers make up the majority of the automotive manufacturing workforce and the share of females was 25–30 percent in 2022 (Moshikaro-Amani, 2023, p. 16). The skill level was much higher at OEMs than in the component manufacturing sector (Mashilo, 2019), enhancing structural power differences between the workforce in OEMs and in the tiers.

Although data on employment security at OEMs and suppliers are hardly available, we can give some indications based on published information. In contrast to the national situation, abusive practices and the share of informal employment seem to be rather rare: 94 percent of the workforce in the automotive sector in the Gauteng region said they have a written contract (according to a non-representative survey, Mashilo, 2019, p. 36). The absence of informal working practices in the sector has been confirmed by our trade union experts, albeit without discussing non-unionized firms or outsourced services such as cleaning or security, for instance.

Data from the National Association of Automobile Manufacturers of South Africa (NAAMSA) covering all OEMs in South Africa show that between 77.2 percent and 84.1 percent of workers were on full-time permanent contracts in the four quarters of 2022 (Table 5). The proportion of permanent employees on part-time contracts remained below 3 %. Workers on casual contracts represented between 1.1 percent and 1.4 percent of the workforce, while workers on temporary contracts were the largest non-permanent category, ranging from 14.5 percent to 18.9 percent throughout 2022. The high variation in the share of non-permanent workers in OEMs during the year is explained with model changes (see below): “*So those are just broad figures. It varies slightly by quarter, […] I see, actually, last year the temporary was only 8 percent, you know, and the permanent was 90 percent. So again, as I said, it varies based on the specific period. I think the reason why the temporary is now higher is because of like a Ford with the launch of a Ranger and the Amarok, so they are getting more people in, to deal with something like that. But I think this year it’s probably 80, 15 […], but last year it was probably 90 and 10 for the temporary.*” (Interview_NAAMSA). In the non-representative survey conducted in the Gauteng region covering both OEMs and suppliers, only 73 percent of workers indicated that they had an indefinite contract (Mashilo, 2019:36). If we consider that the share of suppliers’ employment in the automotive sector is generally higher than that of OEMs, we may estimate that the share of 27 percent flexible employment is due to higher shares of flexible work at suppliers. In line with the rather restrictive legislation and the collective bargaining agreements, however, contract work is at a rather low level. In summary: In both OEMs and suppliers in the South African automotive industry, we find a high proportion of SER employment, albeit more at OEMs than at suppliers.

**Table 5:** Employment by type of contract in all 7 OEMs in South Africa, 2022.

Year	Permanent contracts		Casual contracts	Temporary contracts
	Full-time	Part-time		
	share in percent			
1. Quarter 2022	84.1	0.3	1.1	14.5
2. Quarter 2022	79.9	0.3	1.1	18.7
3. Quarter 2022	79.4	0.3	1.4	18.9
4. Quarter 2022	77.2	2.3	1.3	19.2

Source: Authors’ calculations, Lamprecht, 2023b.

Within OEMs, we also see employment protection practices according to seniority — indicating the implementation of legal segmentation. Additionally, the core workforce at OEMs is protected by employment guarantees given in the collective bargaining agreement (see section 7). This is made possible by structural power differences between OEMs and suppliers, allowing OEMs to avoid retrenchment by in-sourcing or changing contracts with suppliers to make them bear the burden of slack periods. As a union representative stated “*there’s never been a retrenchment in OEMs directly, but indirectly they contribute to retrenchments in the auto sector*” (Interview_GQ). He refers to a case in which redirection of orders to a new supplier led to job shedding at the previous supplier in an attempt to cut costs. “*Like what happened currently in [supplier]. The bulk of the employees have been retrenched, 202, because they could not get the contract from Ford. Due to that, Ford wanted them to be closer to them and they wanted them at a cheaper price*” (Interview_GQ).

Both tier-1 and tier-2 suppliers push for labor flexibility using varied and flexible contract arrangements to be able to respond to OEM demand swings and cost pressures. In a tier-2 enterprise, the expert emphasized that they “*do not want to retrench*” (Interview_CR), but with the increase in fixed-term contracts, the workforce can be adjusted or not retained if demand drops: “*what we have done is we have moved away from permanent employment and we now manage the additional volumes that is required. We will manage on a contract basis and we link that to the lifespan of the contract [with the OEM]*.” (Interview_CR). In this context the expert noted a more recent “*improvement*,” namely the substitution of contract work: “So *we had more than 12 people on our contract basis end of last year. We’ve reduced it down to three.*” (Interview_CR).

This also indicates a practice to use different kinds of temporary work as a kind of probation period to recruit permanent staff, both by tier-1 and tier-2 managers (see comments in Interview_BV). Additionally, however, external factors like load-shedding (power outages) have required flexible work scheduling—another reason why the manufacturer prefers non-standard employment contracts that can be adapted (e.g., by shifting hours or catching up production later) rather than scaling down. Another tier-2 manufacturer has been scaling up its workforce, but doing so also via temporary contracts—albeit at a level of only around 7 %: “*We have about say, 22, 20, because two have just been appointed this week, 20 out of the 300, I would say temps.*” (Interview_BV).

Differences in employment security and quality negotiated in the collective agreements for OEMs and suppliers form part of the segmenting practices at firm level. The implementation of the different working time arrangements contained in the collective agreements is linked to more comfortable shift systems at OEMs. Among suppliers we find a broad variety: “*[…] in the value chain it differs. Some are working 45 h. Yes. It differs in the value chain, but in the OEM it is regulated, is 40 h a week, over and above 40 h it becomes an overtime*” (Interview_VM). Union representation can be decisive to achieve shorter working hours or more comfortable shift systems. A trade unionist reports that in one instance, union input helped change a plant from two 12-h shifts to three 8-h shifts to reduce fatigue and overwork “*So now that shift was implemented to close the gaps whereby they have to put pressure on people to work 12 h then people can work 8 h each and go home. And continue living their lives and do the things that they need to do after work.*” (Interview_ZM).

Moreover, the differences in compensation for short-time working between OEMs and suppliers—as laid down in the collective agreements — result in different reactions to supply-chain disruptions or downturns: At suppliers, short-time work is a rather common reaction, both to deal with last minute changes by OEMs or to keep permanent staff under contract during a lack of demand. “*Many times what would happen, an OEM would say that tomorrow we are not producing… so automatically we are also supposed to stop*,” (Interview_AT) explained a tier-1 manager. In such cases, supplier employees were told not to come in for the cancelled shift, typically without pay for those lost hours. Another tier-1 international component maker acknowledged their short-time working practice to keep permanent staff: “*So that is currently under a strategic review in terms of our employment going forward in the next 12 months, that we will downscale permanent employees based on current volume. And then should volume pick up, we will increase flex labour that will allow us the flexibility to send people home when we do not have volume, but also to employ people when we do have volume, without impacting the permanent staff as it potentially will be now. […] So at the moment, what we do is we are flexing them* [permanent] *based on work requirements and it is no work, no pay. So when we do not need employees, we send them home and when they sit at home, they do not get paid. So the rule is no work, no pay.*” (Interview_SP). This use of short-time working as a practice of ‘scaling labor up or down’ in response to external pressures and uncertainty seems to be rather common as it was also noted by other managers (Interview_AT). It affects not only the flexible workforce, but also core workers. However, differences in the collective agreements result in differences between OEMs and the tiers. As a tier-1 manager noted “*when an OEM would call short time (stop production), they would still pay some 4 h*” (Interview_AT) for that day, while workers at suppliers get nothing.

Finally, problems of compliance with legal regulations or collective agreements may occur both at OEMs and at suppliers. While in the highly unionized OEMs non-compliance is easily contested by collective action, at suppliers that lack effective collective representation, it may directly impact their workers’ employment quality. A trade unionist reported with respect to non-compliance with a collective agreement: *“well, we have our own challenges there [OEM]. Because you’d remember, last year we embarked on a strike that was provoked by employer. Where the employees were supposed to get their bonuses first week of January. But the employer withheld the bonuses and reduced the agreement without even seeking clarity from the trade union.*” (Interview_GQ). The strike, described as “*provoked by [the] employer*,” was a collective effort to insist the company should honor its commitments, thereby safeguarding an earned benefit that contributes to workers’ income security. In contrast, a regional union official observed non-compliance as being common “*in component sectors. …employees should get an increase of 7 %, but the employer will then go and make an application [for exemption] so that the employees should not get an increase*,” even while that supplier passes the cost increase on to their OEM customer (Interview_GQ).

All of this confirms that segmentation practices create large differences in employment security and quality between core and flexible workers across all firms in the automotive sector. In addition, we have shown a deep divide in working conditions between suppliers and OEMs, as flexible work, the risk of redundancy, but also loss of income due to enforced short-time working or unpaid lay-offs, are generally more common in the former — and all of this is compounded by wage and working time differences according to different collective agreements. Furthermore, the generally higher level of union and shop steward representation in OEMs provides a better guarantee that employers will comply with legal or collective regulations than at suppliers, underlining the segmentation of workers’ power resources according to the position of the company.

## 9 Conclusion

The South African automotive industry proves to be a very interesting case with which to analyze the impact of institutional settings and actors’ power resources on influencing segmentation practices at different levels of interaction. A rather universal individual labor law that sharply restricts the use of flexible forms of work is supposed to limit segmenting practices. This is combined with a collective labor law that provides substantial institutional power resources to trade unions allowing them to build up associational power, comprehensive bargaining structures, and effective workplace representation in the production sites. However, despite this institutional framework we find pronounced segmentation practices affecting employment security and quality in South Africa’s automotive industry. The idea of an ‘enclave’ may only be supported with respect to the possibly worse conditions on the national labor market — but not in the sense of an entirely well-protected and equalized labor market.

Firms exploit all available legal contract forms to maximize flexibility in order to cope with fluctuations in demand. These practices are to be found across the sector, both at major vehicle producers (OEMs) and suppliers, although the former generally offer more secure, standard employment, while supplier firms more often rely on temporary or other non-standard contracts. The OEM–supplier divide is further reinforced by dualized collective bargaining arrangements. Wages negotiated by OEMs on the national level via a dedicated National Bargaining Forum are much higher than in the most relevant sector-specific agreement involving suppliers (MIBCO, 2023). Additionally, we see major differences concerning agreed working time, social benefits, and pay during short-time work, with consequences for overtime, bonuses, and other working conditions.

These disparities reflect underlying power dynamics in the production network as suggested by the GVC and GNP approach and underscore that labor market segmentation theory should more strongly consider the position of the firm in the production network to understand segmenting practices. Overall, managers’ and trade unionists’ assessments of how changes in demand from the OEMs are handled indicate that the difference in structural power between OEMs and suppliers is a driver for the named segmentation practices — albeit this also includes particular differences due to management strategies. Additionally, our results indicate that the differences in employers’ structural power resources more or less ‘reproduce’ differences in the structural — and possibly also organizational — power resources of trade unions and workers’ representatives in the different workplaces. This limits measures to counteract precarious employment conditions in the more dependent or vulnerable firms of the lower tiers. We may therefore summarize that even South Africa’s robust labor laws and strong unions cannot fully counteract the structural power imbalances along the value chain as employers’ cost-cutting logics remain central to competition, leading to deteriorating labor standards at suppliers.

By integrating segmentation theory with the Global Production Network (GPN) approach and power resource theory, the study offers a nuanced understanding of segmentation practices. The South African case illustrates how competitive pressures can erode even strict local labor standards, explaining why segmentation persists despite robust formal frameworks and a rather powerful labor movement. Thus, the findings underscore the need for multi-level efforts to mitigate entrenched segmentation in global production networks.

The study’s scope is limited to structural and institutional drivers; factors like race and gender, though acknowledged, are not examined in depth. For instance, persistent racial and gender inequalities — e.g. Black workers concentrated in lower-tier roles and few women in management — are noted as a broader context rather than analyzed in detail. Future research could examine how these social cleavages intersect with structural segmentation, or investigate similar patterns in other sectors and regions.

Several other questions also require further research: To what extent do segmentation practices within increasingly complex and transnational production networks act to reinforce, destabilize, or transform the normative-legal architecture of employment segmentation? In light of growing institutional plurality, organizational flexibilization, and the juridical fragmentation of labor standards across organizational and territorial boundaries, further empirical and theoretical inquiry is needed into the dialectical relationship between formal regulatory frameworks and the everyday enactment of segmentation practices in firms. Such inquiries promise not only to shed light on the mechanisms of labor market stratification but also on the evolving nature of institutional control and regulatory governance in contemporary capitalism. Within this discourse, more research on workers’ agency and their power resources is also needed. How could workers’ organizations better deal with low structural power resources due to the market position of their employer? How could comprehensive bargaining structures be achieved in order to create more equal employment and pay structures within an industry? We hope that the case study of the South African automotive industry inspires further research on these topics.

## Data Availability

The interview transcripts analyzed in this article are not publicly available, as interviewees granted consent for use within the scope of the original research project only and not for secondary use in other projects. Requests for access to the interview transcripts may be directed to Andrea Schaefer andrea.schaefer@uni-bremen.de. The data on labor law norms (see Carlino et al. 2024) and coding rules for indices and functions (see Schäfer et al. 2024a,b,c) used in this study are publicly available via the WeSIS platform (https://wesis.org/) under the topic “Social Policies” and sub-topic “Labour and Labour Market”. This study uses WeSIS indicators based on the CBR-LRI dataset, supplemented with datapoints from WoL (see Release Date: data version 2, updated version 2024).

## Footnotes

1 Segmentation practices are understood as everyday formal routines that actors (at industry and workplace levels) implement, guided by institutional frameworks and influenced by their power resources. The accumulation of these segmentation practices leads to specific segmenting patterns.

2 The overrepresentation of these groups in precarious labor market segments has particularly been analyzed by *feminist contributions* (see, e.g., Rubery and Piasna, 2016; Grimshaw et al., 2017, p. 2).

3 This has been confirmed by (Barrientos, 2013, p. 1969) who argued that commercial chains linking firms are replicated through labor chains linking producers, contractors, and workers. Particularly within labor contracting, a continuum of labor relations coexists, from better to worse.

4 Other very inspiring (comparative) case studies on the automotive industry in different countries (Jürgens and Krzywdzinski, 2016; Barnes et al., 2017; Krzywdzinski, 2017) analyze working conditions in more detail, but do not focus on regional value chains.

5 Special economic zones (SEZs) and Industrial Development Zones (IDZ) offer tax incentives and supportive policies, providing favourable conditions for manufacturing operations (https://www.statssa.gov.za/?page_id=1021&id=city-of-tshwane-municipality).

6 We excluded three interviews: one interviewee withdrew their consent for the use of their material; two interviews were conducted with union coordinators from other branches, such as security and cleaning.

7 Source: https://www.gov.za/sites/default/files/gcis_document/201409/act66-1995labourrelations.pdf; downloaded April 28, 2025.

8 Source: https://www.ccma.org.za/labourlaws/new-minimum-wage-and-maximum-threshold/; downloaded April 17, 2025.

9 Source: https://www.gov.za/sites/default/files/gcis_document/201409/a75-97.pdf; downloaded April 28, 2025.

10 The flexibility function summarizes the following indicators: fixed-term contracts are allowed only for work of limited duration; maximum duration of fixed-term contracts; agency work is prohibited or strictly controlled; part-time workers have the right to equal treatment; dismissal costs for part-time workers are proportional; fixed-term workers have the right to equal treatment, and agency workers have the right to equal treatment (calculation based on data from www.wesis.org).

11 See https://oica.net/wp-content/uploads/2025/11/Passenger-Cars-2022.pdf, downloaded August 21, 2025.

12 Exchange rate 2023: 1 USD = 18.5 R on average, see: https://www.dollarfx.org/South-African-Rand/2023
